# Effect of body position on cerebral perfusion: a comparison of supine and seated positions assessed using conventional and upright CT perfusion

**DOI:** 10.1186/s41747-026-00747-6

**Published:** 2026-06-08

**Authors:** Katsuhiro Mizutani, Yoshitake Yamada, Minoru Yamada, Yoichi Yokoyama, Kenzo Kosugi, Keisuke Yoshida, Satoshi Takahashi, Takenori Akiyama, Masahiro Toda, Masahiro Jinzaki

**Affiliations:** 1 Department of Neurosurgery, Keio University School of Medicine, Tokyo, Japan; 2 Department of Radiology, Keio University School of Medicine, Tokyo, Japan; 3 Department of Neurosurgery, Mihara Memorial Hospital, Gunma, Japan

**Keywords:** Adaptation (physiological), Cerebrovascular circulation, Multidetector computed tomography, Seated position, Supine position

## Abstract

**Objective:**

Postural changes influence systemic hemodynamics and may affect cerebral perfusion; however, quantitative evaluation remains limited *in vivo* under physiological gravitational conditions. We aimed to assess posture-dependent alterations in cerebral perfusion using upright multidetector computed tomography perfusion (CTP), with whole-brain cerebral blood flow (CBF) as the primary outcome.

**Materials and methods:**

This was a single-center observational study of healthy volunteers approved by the institutional review board. Eighteen volunteers underwent CTP imaging in both the supine and seated positions on the same day using two 320-row multidetector CT scanners with identical systems. Whole-brain CBF was defined as the primary endpoint. Mean transit time (MTT) and cerebral blood volume (CBV) were evaluated as secondary outcomes. Paired comparisons were performed between postures, and exploratory analyses assessed regional changes and interindividual variability.

**Results:**

Whole-brain CTP-derived CBF was significantly higher in the seated position than in the supine position (32.9 ± 6.0 [mean ± standard deviation] *versus* 29.2 ± 5.6 mL/100 g/min; *p* = 0.010), representing a 1.15 ± 0.19-fold increase. CTP-derived MTT was significantly shorter in the seated position (4.25 ± 0.61 *versus* 4.63 ± 0.72 s; *p* = 0.035), and CTP-derived CBV showed a modest but significant increase (2.23 ± 0.16 *versus* 2.16 ± 0.17 mL/100 g; *p* = 0.044). Regional perfusion changes were consistent across brain segments with interindividual variability in postural responses.

**Conclusions:**

Upright CTP detected posture-associated differences in whole-brain CTP-derived perfusion metrics, suggesting potential utility for investigating physiological and pathological alterations in cerebral hemodynamics associated with postural change.

**Relevance statement:**

Upright CTP enables physiological evaluation of posture-related cerebral hemodynamics under gravitational loading and may provide new insights into cerebral circulation in both health and disease.

**Key Points:**

- Whole-brain cerebral blood flow increased by 15% in the seated position compared with supine using upright CT perfusion.
- Mean transit time shortened by 7% and cerebral blood volume increased modestly by 4% in the seated position.
- Perfusion responses showed marked interindividual variability, suggesting different physiological adaptations to posture.

**Graphical Abstract:**

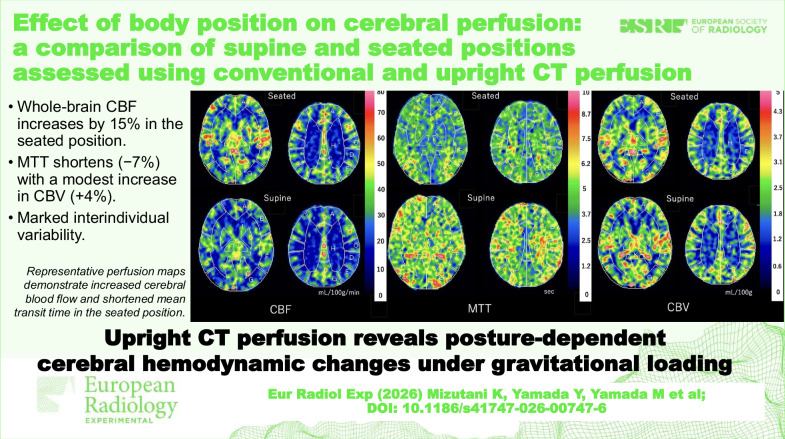

## Background

The human brain is a highly energy-intensive organ. Although it comprises only approximately 2% of total body mass, it consumes more than 10% of total cardiac output [1]. To meet this substantial metabolic demand, the body has developed regulatory mechanisms to maintain adequate cerebral perfusion [2–4]. Cerebral blood flow (CBF) is widely recognized as a key quantitative indicator of brain perfusion [2–4]. Cerebral perfusion pressure, defined as the difference between mean arterial pressure (MAP) and intracranial pressure, represents the driving force for CBF [2, 3, 5].

Postural changes can significantly affect systemic hemodynamics and cerebral circulation. When transitioning from a supine to a seated or upright position, blood is redistributed to the lower part of the body, generating a hydrostatic gradient between the heart and the cranial circulation [6–8]. These physiological changes may influence cerebral perfusion; however, despite their relevance, relatively few studies have directly evaluated posture-related alterations in cerebral perfusion *in vivo*. Previous investigations using positron emission tomography (PET), xenon-based techniques, and transcranial Doppler have reported inconsistent findings and were often limited by restricted spatial resolution or regional coverage [9–12].

Recently, the development of upright multidetector computed tomography (CT), which allows imaging under gravitational loading conditions [13, 14], has enabled objective evaluation of posture-related changes in various organs, including the brain [13, 15] and cerebrovascular system [13, 16]. However, quantitative assessment of cerebral perfusion using upright CT perfusion (CTP) remains limited.

We hypothesized that upright multidetector CTP would enable detection of posture-dependent alterations in cerebral perfusion, with whole-brain CBF as the primary outcome. Therefore, in this study, we aimed to compare whole-brain CTP-derived CBF between the supine and seated positions using upright CTP. Secondary analyses included evaluation of CTP-derived mean transit time (MTT), cerebral blood volume (CBV), and interindividual variability in perfusion responses.

## Methods

### Participants

This was a single-center retrospective analysis of prospectively collected data, approved by the institutional review board (approval no. 20221150, January 12, 2023). We retrospectively analyzed data from 20 healthy volunteers (10 men and 10 women) who had been enrolled in a previous prospective study (approval no. 20180035; UMIN-CTR: UMIN000032999, June 15, 2018). The original prospective study was designed to comprehensively evaluate the effects of postural change on intracranial hemodynamics, including arterial, tissue, and venous components, under physiological conditions. Written informed consent was obtained from all participants in the original prospective study. The requirement for additional informed consent for this retrospective analysis was waived by the ethics committee. The study was conducted in accordance with the ethical standards of the 1964 Declaration of Helsinki and its later amendments. Inclusion criteria were: age between 30 and 80 years; estimated glomerular filtration rate ≥ 60 mL/min/1.73 m²; and no history of smoking, systemic disease, or ongoing medical treatment. Exclusion criteria were: pregnancy or suspected pregnancy; bronchial asthma; or a history of adverse reactions to iodinated contrast media.

### Study protocol

Supine and seated CT scans were performed on the same day, with a 1-h interval between scans, from August 25, 2018, to February 15, 2019. For imaging in each posture, we used a supine 320-detector row multidetector CT scanner (Aquilion ONE; Canon Medical Systems) and an upright 320-detector row multidetector CT scanner (Aquilion ONE GENESIS edition; Canon Medical Systems), which was adapted from the conventional supine model and demonstrated comparable performance [13]. The order of scans was randomized in a 1:1 ratio. Before image acquisition, participants remained in each position (supine or seated) for at least 15 min to ensure hemodynamic stabilization. Heart rate, systolic arterial pressure, diastolic arterial pressure, and MAP were recorded before and after each scan using noninvasive forearm cuff measurements. In upright scanning, participants were seated, and their heads were firmly stabilized using a dedicated fixation system, including a forehead strap attached to the chair.

### CTP scan protocol

Following a test bolus injection (10 mL of iopamidol (Oypalomin; Fuji Pharma Co., Ltd.) with an iodine concentration of 370 mgI/mL at 5 mL/s, followed by 20 mL of saline), the scan timing was determined based on the arrival of contrast material at the carotid bulb, and computed tomography perfusion was performed using 50 mL of the same contrast agent at the same injection rate, followed by an additional 20 mL of saline. The perfusion scan was performed with whole-brain coverage using a 320-detector row CT scanner, enabling volumetric acquisition of the entire brain in a single rotation. An unenhanced mask image was first acquired with a 1-s scan. Subsequent imaging was performed at the following time points after contrast injection: 0, 2, 4, 6, 8, 10, 12, 14, 16, 18, 20, 23, 26, 29, 32, and 60 s. Scan parameters were as follows: 0.5 mm slice thickness without overlap, 24 cm field of view (voxel size: 0.46 × 0.46 × 0.5 mm³), 80 kV, 350 mA for the mask image, 150 mA for intermittent acquisitions, and 1-s rotation time. Images were reconstructed using an adaptive iterative reconstruction technique (FC43 kernel; Canon Medical Systems). The estimated effective radiation dose was 3.66 mSv in the seated position and 3.42 mSv in the supine position. These values were calculated using the dose-length product: 1,742 mGy·cm for the seated position and 1,628.2 mGy·cm for the supine position, applying the standard conversion coefficient for head CT (0.0021 mSv/mGy·cm).

### Cardiovascular parameters

Heart rate, systolic arterial pressure, diastolic arterial pressure, and mean arterial pressure (MAP) were compared between the supine and seated positions, using the average values recorded before and after each CT scan. Cardiovascular parameters that showed significant postural differences were subsequently used in later analyses to explore their associations with posture-induced changes in cerebral perfusion.

### Postural influence on brain perfusion

Perfusion parameters in the supine and seated positions were compared within the same participants. Perfusion maps were generated using the CTP workstation console (VES version 6.4.2). The analysis was primarily automated, with the exception of the arterial input function (left proximal M1 segment of the middle cerebral artery) and venous outlet function (superior sagittal sinus), which were manually selected by an experienced interventional neuroradiologist (> 10 years of experience in cerebrovascular disease management). For each participant, the arterial and venous input locations were selected at the same anatomical segment in both supine and seated scans to ensure intra-individual consistency. CBF, CBV, and the MTT maps (512 × 512 matrix × 320 slices, 0.5 mm thickness) were generated using the singular value decomposition plus deconvolution algorithm. The average CTP-derived CBF, CBV, and MTT values for each brain region were calculated using 3DSRT Neuro, version 1.0.7 (PDRadiopharma Inc.) [17]. The software reduced each perfusion dataset to standardized slices at 2-mm intervals for regional segmentation. These standardized maps were automatically segmented into 12 brain regions: callosomarginal, precentral, central, parietal, angular, temporal, posterior cerebral, pericallosal, lenticular nucleus, thalamus, hippocampus, and cerebellum. The whole-brain perfusion values were calculated as the unweighted mean of the 12 predefined brain segments. Both whole-brain and regional perfusion values were compared between the supine and seated positions. Furthermore, associations between the aforementioned cardiovascular parameters and the rate of change in each perfusion parameter during postural transition were evaluated, along with other individual characteristics such as age, height, and weight.

### Statistical analysis

Continuous variables are presented as mean ± standard deviation. Paired *t*-tests were used to compare perfusion parameters and physiological variables between the seated and supine positions. Normality of the data distribution of paired differences was assessed using visual inspection of histograms and Q–Q plots, and no substantial deviation from normality was observed. For the primary and secondary endpoints, the mean paired difference and corresponding 95% confidence interval were calculated. Effect sizes for paired comparisons were estimated using Cohen’s dz. Between-group differences were evaluated using two-sample *t*-tests, and associations between continuous variables and perfusion changes were assessed using Pearson’s correlation coefficients. Subgroup analyses were performed to further explore the observed findings and were considered exploratory; therefore, no correction for multiple comparisons was applied. These results should be interpreted descriptively. Statistical significance was defined as *p* < 0.05. Statistical analyses were performed using Python (version 3.10.13) with standard scientific libraries.

## Results

### Participants

**Fig. 1:**
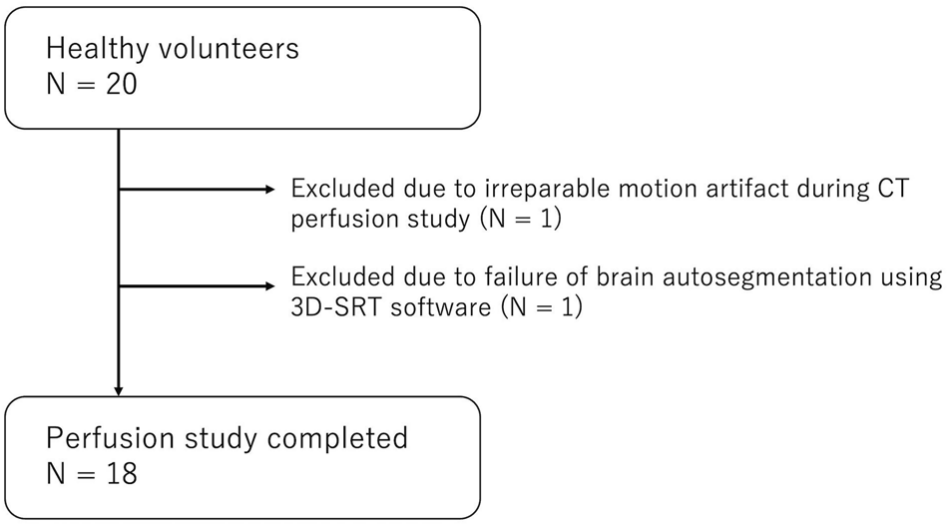
Of the 20 volunteers initially enrolled, two were excluded (motion artifact, *n* = 1; failed automated segmentation, *n* = 1). The final analysis included 18 participants

Of the 20 volunteers initially enrolled, one was excluded due to an irrecoverable motion artifact, and in another participant, the 3D-SRT software failed to accurately segment the predefined 12 brain regions. Consequently, the final perfusion study was completed with 18 participants (nine men and nine women) (Fig. 1). The age of the participants was 43.4 ± 7.4 years (mean ± standard deviation), with a mean height and weight of 163.9 ± 6.4 cm and 59.6 ± 9.4 kg, respectively.

### Cardiovascular parameters

The heart rate remained stable during postural changes, with values of 73.9 ± 12.9 beats per min and 73.5 ± 14.8 beats per min in the seated and supine positions, respectively (*p* = 0.824). Diastolic arterial pressure and MAP were significantly higher among participants in the seated position (diastolic arterial pressure: 81.5 ± 10.1 mmHg *versus* 77.5 ± 11.9 mmHg*, p* = 0.011; MAP: 94.9 ± 10.7 mmHg *versus* 90.8 ± 12.4 mmHg*, p* = 0.004). Although systolic arterial pressure was higher in the seated position (121.8 ± 13.5 mmHg *versus* 117.4 ± 14.5 mmHg), this difference did not reach statistical significance (*p* = 0.054).

### Postural influence on brain perfusion

**Fig. 2:**
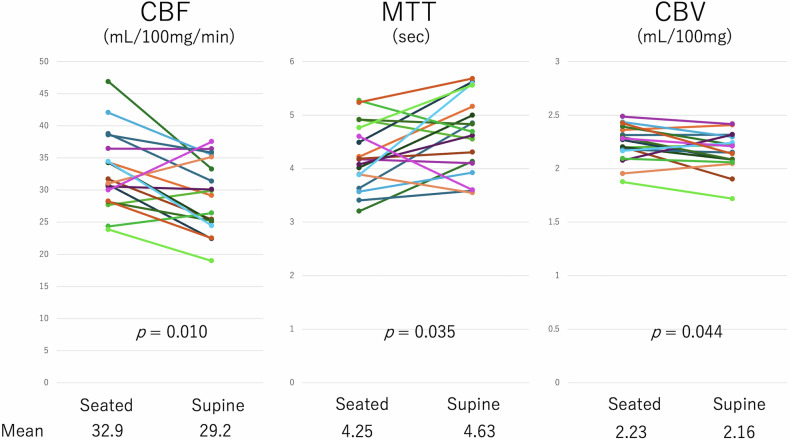
Individual changes in whole-brain cerebral perfusion parameters (cerebral blood flow (CBF), mean transit time (MTT), and cerebral blood volume (CBV) during postural transition. Each line represents an individual participant, connecting perfusion values measured in the supine and seated positions. Differences were assessed using paired *t*-tests

The results of the perfusion analysis under different postural conditions are presented in Fig. 2 and Table 1. The mean whole-brain CTP-derived CBF was significantly higher in the seated position than in the supine position (32.9 ± 6.0 mL/100 g/min *versus* 29.2 ± 5.6 mL/100 g/min), representing a 1.15 ± 0.19-fold increase. The mean paired difference was 3.75 mL/100 g/min (95% CI, 1.03–6.46; *p* = 0.010). Similarly, the mean whole-brain CTP-derived MTT was shorter in the seated position (4.25 ± 0.61 s *versus* 4.63 ± 0.72 s), reflecting a 0.93 ± 0.15-fold decrease. The mean paired difference was -0.38 s (95% CI, -0.68 to -0.08; *p* = 0.035). The whole-brain CTP-derived CBV was also slightly higher when seated (2.23 ± 0.16 mL/100 g *versus* 2.16 ± 0.17 mL/100 g), representing a 1.04 ± 0.06-fold increase. The mean paired difference was 0.07 mL/100 g (95% CI, 0.00–0.13; *p* = 0.044). The effect sizes for the paired comparisons were moderate for CBF (Cohen’s dz = 0.69) and MTT (dz = 0.53), and small-to-moderate for CBV (dz = 0.49). These postural effects on brain perfusion parameters were consistent across all brain segments and between the left and right brains (Table 2). In addition, the rates of change in CTP-derived CBF, MTT, and CBV were consistent between female and male participants. No significant associations were found between participants’ cardiovascular and demographic characteristics and the rate of change in the whole-brain CTP-derived CBF, MTT, and CBV. Further details are provided in Supplementary Figs. S1 and S2.

**Table 1:** Demographics and perfusion change ratios (seated/supine)

Case	Sex	Age (decade)	Height (cm)	Weight (kg)	CBF change (seated/supine)	MTT change (seated/supine)	CBV change (seated/supine)	Pattern
1	M	50 s	168	55	1.41	0.78	1.08	I
2	F	40 s	168	63.1	1.41	0.69	0.97	I
3	M	50 s	173	67	1.38	0.80	1.09	I
4	M	50 s	171	68	1.36	0.80	1.06	I
5	M	30 s	166	60.5	1.26	0.86	1.10	I
6	F	30 s	157	45	1.26	0.92	1.13	I
7	M	30 s	168.5	55	1.25	0.97	1.16	
8	F	50 s	154	46	1.23	0.75	1.00	I
9	F	40 s	154	59	1.19	0.91	1.06	I
10	M	40 s	167.5	57	1.18	0.82	0.98	I
11	F	40 s	158	62	1.12	1.02	1.10	II
12	F	50 s	160.2	55.3	1.08	0.95	1.01	II
13	F	30 s	154	85	1.02	0.88	0.90	
14	F	30 s	160	48	1.00	1.02	1.03	II
15	M	40 s	170	68.5	0.93	1.08	0.99	II
16	M	40 s	171	65	0.92	1.12	1.02	II
17	M	40 s	164	57	0.89	1.10	0.96	II
18	F	50 s	165.5	56	0.80	1.28	1.03	

Pattern I: responsive group, Pattern II: non-responsive group

*CBF* Cerebral blood flow, *MTT* Mean transit time, *CBV* Cerebral blood volume

**Table 2:** Average change rate (seated/supine positions) of perfusion parameters during postural changes in each brain segment

	Regional CBF		Regional MTT		Regional CBV	
	Right	Left	Right	Left	Right	Left
Mean across all segments (per hemisphere)	1.15	1.15	0.95	0.92	1.05	1.03
Callosomarginal	1.15	1.15	0.92	0.93	1.04	1.03
Precentral	1.16	1.15	0.95	0.94	1.06	1.04
Central	1.13	1.15	0.96	0.92	1.05	1.03
Parietal	1.16	1.17	0.96	0.92	1.07	1.05
Angular	1.15	1.17	0.97	0.93	1.05	1.05
Temporal	1.15	1.16	0.97	0.93	1.07	1.05
Posterior cerebral	1.14	1.14	0.96	0.93	1.04	1.04
Pericallosal	1.13	1.15	0.94	0.93	1.03	1.03
Lenticular nucleus	1.15	1.16	0.91	0.89	1.02	1.00
Thalamus	1.12	1.14	0.92	0.93	1.00	1.00
Hippocampus	1.17	1.14	0.94	0.91	1.06	1.02
Cerebellum	1.15	1.17	0.94	0.87	1.05	1.02

*CBF* Cerebral blood flow, *MTT* Mean transit time, *CBV* Cerebral blood volume

**Fig. 3:**
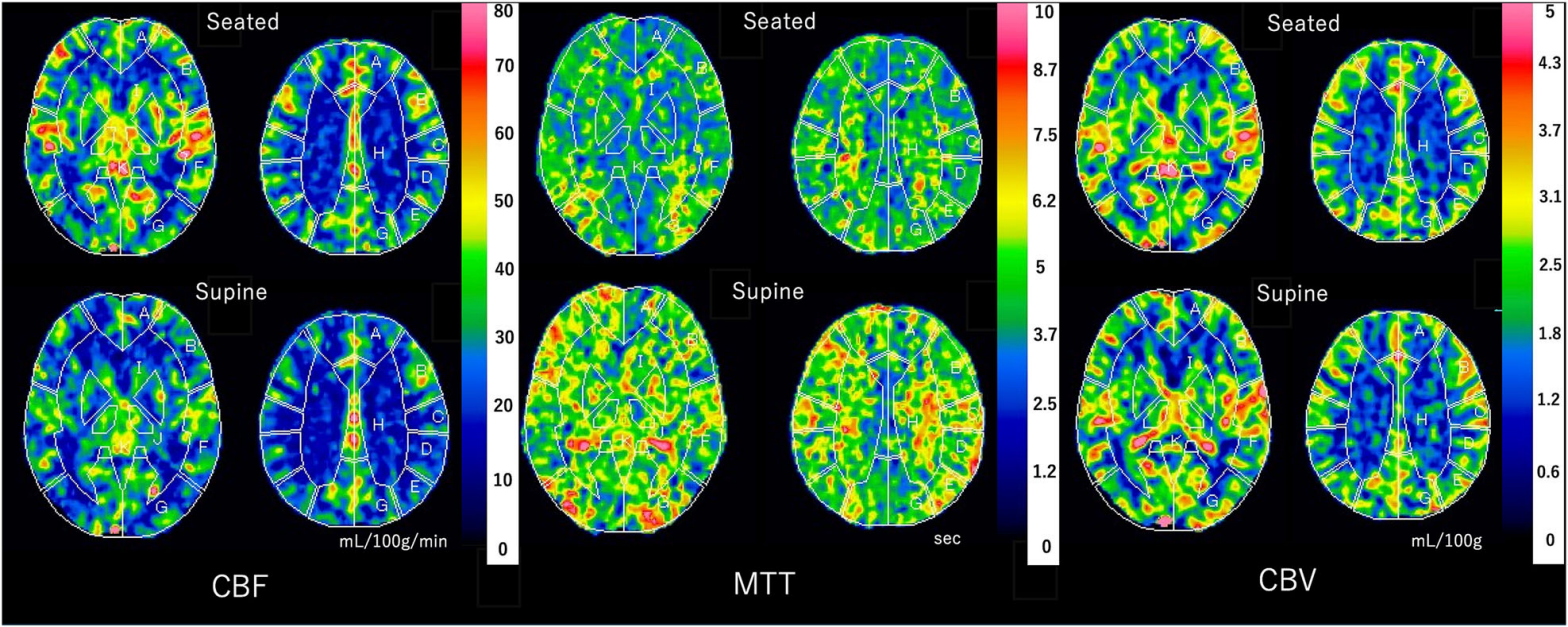
Representative perfusion maps of Pattern I (Case 10, male in his 40 s). Two representative axial levels from standardized perfusion maps of whole-brain cerebral blood flow (CBF), mean transit time (MTT), and cerebral blood volume (CBV) are shown for the supine and seated positions. Maps were automatically generated and segmented using 3DSRT Neuro into predefined brain regions: A, callosomarginal; B, precentral; C, central; D, parietal; E, angular; F, temporal; G, posterior cerebral; H, pericallosal; I, lenticular nucleus; J, thalamus. In this pattern, CBF increased and MTT decreased in the seated position

**Fig. 4:**
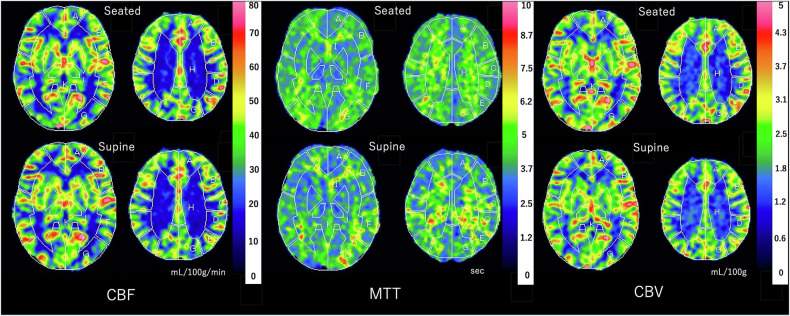
Representative perfusion maps of Pattern II (Case 14, female in her 30 s). Two representative axial levels from standardized perfusion maps of whole-brain cerebral blood flow (CBF), mean transit time (MTT), and cerebral blood volume (CBV) are shown for the supine and seated positions. Maps were segmented in the same manner as in Fig. 3. In this pattern, perfusion parameters remained relatively stable between postures

To further illustrate interindividual variability, an exploratory *post hoc* subanalysis was performed. Participants were descriptively categorized according to relative changes in CTP-derived CBF (> 15% increase) and CTP-derived MTT (> 7% decrease), using the average observed changes. This categorization was not predefined in the study protocol. Based on these criteria, two descriptive patterns of perfusion change were observed (Table 1): The first group (Pattern I, *n* = 9) exhibited an increase in CTP-derived CBF (1.30 ± 0.09-fold on average), a shortening of CTP-derived MTT (0.81 ± 0.07-fold on average), and a minimal change in CTP-derived CBV (1.05 ± 0.06-fold on average) in the seated position. The second group (Pattern II, *n* = 6) showed relatively stable values across all three parameters: CTP-derived CBF (0.99 ± 0.09-fold on average), CTP-derived MTT (1.05 ± 0.06-fold on average), and CTP-derived CBV (1.02 ± 0.05-fold on average). The remaining three participants exhibited perfusion patterns that differed from those of the two patterns (Table 1). The pattern-specific perfusion parameter changes are shown in Supplementary Fig. S3 (Pattern I) and Supplementary Fig. S4 (Pattern II). Aside from the difference in CTP-derived CBF and MTT, no significant differences were observed in participants’ background characteristics between the two patterns (Table 3). Representative perfusion images for each pattern are presented in Fig. 3 (Pattern I) and Fig. 4 (Pattern II).

**Table 3:** Comparison of patient backgrounds between Group I and Group II

	Pattern I (*n* = 9) (Mean ± SD)	Pattern II (*n* = 6) (Mean ± SD)	Remaining cases (Mean ± SD)	*p*-value (Comparison between Patterns I and II)
Age (years)	45.0 ± 8.0	42.3 ± 5.2	41.0 ± 10.8	0.448
Height (cm)	164.3 ± 7.3	163.9 ± 5.5	162.7 ± 7.7	0.903
Weight (kg)	57.8 ± 8.2	59.3 ± 7.4	65.3 ± 17.0	0.727
Diastolic blood pressure difference (seated-supine) (mmHg)	2.72 ± 6.16	4.17 ± 5.12	7.50 ± 6.04	0.630
Mean arterial pressure difference (seated-supine) (mmHg)	3.19 ± 3.40	3.33 ± 6.82	8.50 ± 4.81	0.962
CBF change rate (seated/supine)	1.30 ± 0.09	0.99 ± 0.09	1.02 ± 0.22	< 0.001
MTT change rate (seated/supine)	0.81 ± 0.07	1.05 ± 0.06	1.04 ± 0.21	< 0.001
CBV change rate (seated/supine)	1.05 ± 0.06	1.02 ± 0.05	1.03 ± 0.13	0.251

*p* < 0.05 was considered statistically significant. The remaining cases are presented for reference only and were not included in the statistical comparison

*CBF* Cerebral blood flow, *MTT* Mean transit time, *CBV* Cerebral blood volume

## Discussion

In this study, upright CT perfusion demonstrated a significant increase in whole-brain CTP-derived CBF in the seated position compared with the supine position, accompanied by a shortening of CTP-derived MTT and a modest increase in CTP-derived CBV.

In the present study, diastolic blood pressure and mean arterial pressure were slightly higher in the seated position, whereas heart rate remained unchanged. These changes in blood pressure are broadly consistent with previous reports [18–20], while heart rate is generally reported to increase slightly in the seated position [21, 22]. This discrepancy may be attributed to the relatively small sample size or the influence of the CT scanning environment, which may include mild anxiety compared with a resting physiological setting. These cardiovascular changes may partly contribute to posture-related alterations in cerebral perfusion.

Positional effects on cerebral perfusion have been described in numerous studies [9–12, 23–25]; however, they remain incompletely understood due to technical and methodological challenges in assessing dynamic physiological changes during postural transitions [2]. Although CBF is widely used as a key parameter of cerebral perfusion [2–4], it does not fully capture cerebral metabolism, which is also influenced by oxygen delivery, including oxygen extraction and arterial oxygen content [26]. Furthermore, CBF responds rapidly to physiological fluctuations, often within seconds [23, 24], whereas imaging modalities such as PET, single-photon emission computed tomography, and CTP lack sufficient temporal resolution to capture these transient changes. Importantly, CBF is influenced by multiple interacting factors, including arterial pressure, venous outflow, and PaCO_2_ [2, 27], which change simultaneously during postural transitions and cannot be independently controlled or measured with current imaging techniques. Therefore, CBF does not fully represent cerebral perfusion, as it does not capture the complex interplay between flow, metabolism, and oxygen delivery. Moreover, it remains difficult to accurately infer the underlying physiological mechanisms associated with postural changes based solely on CBF measurements. Therefore, the present findings should be interpreted as posture-associated “static” changes in CTP-derived perfusion metrics rather than direct dynamic observations of true cerebral blood flow or the fundamental nature of cerebral perfusion.

The CTP-derived perfusion changes observed in the present study, particularly the increase in CTP-derived CBF, are intriguing. Postural changes in cardiovascular hemodynamics may partly contribute to these observations. In the upright posture, if MAP at the level of the brachial artery remains unchanged, it is expected to decrease by approximately 20 mmHg at the level of the brain [6, 8]. Similarly, intracranial pressure, which reflects the venous outlet pressure [27, 28], also decreases due to reduced hydrostatic venous pressure in the brain. Compared to arteries, veins have thinner, weaker, and more compliant walls. Consequently, excessive negative pressure can cause venous collapse [16, 29, 30], leading to a relatively smaller reduction in intracranial pressure than in MAP at the level of the brain [8]. Thus, cerebral perfusion pressure in the seated position is expected to decrease relative to that in the supine position [31, 32], despite the minor increase in forearm MAP observed in the present study. Cerebral perfusion pressure represents the pressure gradient driving cerebral blood flow, in accordance with the fundamental Starling principle. We hypothesized that CBF may increase to compensate for this reduction in cerebral perfusion pressure, ensuring sustained energy and oxygen supply to the brain.

In addition, approximately 10% of blood volume is redistributed to the lower body in the upright position [7], resulting in a reduction in effective circulating blood volume [2]. Under such conditions, maintaining cardiac output with reduced circulating volume may require increased flow and shortened transit time at the tissue level, which could partly explain the observed increase in CTP-derived CBF and shortening of MTT. In the present study, CBV showed a slight increase. Because CBF, MTT, and CBV are interrelated through the central volume principle [33], this finding may reflect the combined effects of changes in flow and transit time. Another possible mechanism involves cerebrospinal fluid redistribution. In the upright position, cerebrospinal fluid shifts caudally under gravitational influence, potentially reducing intracranial cerebrospinal fluid volume [15]. According to the Monro–Kellie doctrine [34], intracranial volume is maintained as a constant sum of brain tissue, blood, and cerebrospinal fluid; therefore, a reduction in cerebrospinal fluid volume may be accompanied by a compensatory increase in intracranial blood volume.

Conversely, in the second pattern (Pattern II), the imbalance between arterial and venous pressure during postural transition may be minimal, resulting in relatively stable perfusion parameters. The reason for these different brain perfusion patterns remains unclear, as no significant differences were observed in the hemodynamic or physical parameters between the patterns. Three additional participants showed minor variations. These variations may reflect how a healthy human brain responds differently to postural changes. However, these subgroup patterns should be interpreted as exploratory findings derived from a small sample size and identified in a *post hoc* manner, and therefore do not represent definitive physiological classifications. Although speculative, comparing individuals with different response patterns may provide a useful framework for generating hypotheses regarding factors that influence posture-related perfusion changes. Further studies with larger cohorts are required to validate these observations and to clarify the underlying mechanisms.

In summary, individual differences in cardiac functional reserve, vascular anatomy, and physiological conditions may influence postural perfusion patterns. Although direct, simultaneous measurement of MAP at the level of the brain, intracranial pressure, PaCO₂, and arterial oxygen content in healthy humans is currently not feasible, our findings highlight the need for further investigation into posture-related perfusion dynamics.

Previous literature has assessed the changes in cerebral perfusion using other modalities, including H_2_(15)O PET [9], Xenon-133 inhalation with scintillation detectors [10, 12], 99 m Tc-hexamethyl-propylene amine oxime-single-photon emission computed tomography [11] and transcranial Doppler echogram [25]. However, only a few studies have reported brain perfusion in a seated position in healthy participants [9, 12], and the effect of posture on cerebral perfusion remains poorly established in the literature. In those studies that employed static postural conditions similar to those in this study, the seated position was associated with either a small, nonsignificant reduction [9] or a nonsignificant increase [12] in the CBF in the cerebral hemispheres compared to the supine position. Inconsistencies across studies may have resulted from differences in the study design. Earlier studies measured only CBF, often using manually drawn regions of interest and in a limited number of brain regions, relying on modalities with lower resolution [9, 12]. In contrast, our study employed automated whole-brain segmentation across standardized CT slices, allowing objective quantification of regional CTP-derived CBF, MTT, and CBV. Individual variability in postural perfusion responses may partly explain inconsistencies in previous reports, many of which included fewer than 10 healthy participants and were not designed to assess interindividual differences. A major strength of this study is the use of upright multidetector CT, which enables perfusion imaging under physiological gravitational conditions. These findings highlight the potential utility of upright CT perfusion for investigating posture-related cerebral hemodynamics and may provide a framework for future studies examining physiological and pathological alterations associated with postural change. Compared with upright single-photon emission computed tomography or PET, upright multidetector CT may provide a practical alternative approach for evaluating posture-related cerebral perfusion. As this technology becomes more widely adopted, its potential utility may extend beyond research settings to clinical applications.

This study had certain limitations. First, the interpretation of posture-related changes in CBF is inherently limited by the complex and dynamic interplay of multiple physiological factors. Therefore, the findings should be considered as CTP-derived perfusion metrics rather than direct measures of absolute cerebral blood flow. Second, quantitative analyses of CT perfusion are considered less reliable than those of PET [35] or Xenon CT [17], and the current values may not perfectly reflect the true values for each participant. However, most findings in this study were derived from relative comparisons between the seated and supine positions in the same individual. CT perfusion remains useful for qualitative analyses and interindividual comparisons, even if it lacks precision for absolute quantification [17]. Our findings should be interpreted in this context and warrant validation using more quantitative reference methods. Third, the arterial input was set in the left proximal M1 segment of the middle cerebral artery. In general, large proximal arteries, including the internal carotid artery and middle cerebral artery, are recommended for arterial input selection [36]. In this study, the proximal M1 segment was chosen because it has a relatively straight course, facilitating stable and reproducible arterial input placement. This was particularly important to ensure consistent positioning within the same vascular segment across both supine and seated conditions. Ideally, the calculation of tissue perfusion parameters requires a time-density curve of the upstream arterial input. Consequently, this analysis may have produced relatively inaccurate values in the posterior circulation and right hemisphere. Fourth, PaCO_2_ and other physiological parameters were not measured in this study and may have influenced cerebral perfusion metrics. As noted above, such factors can substantially affect and further limit physiological interpretation. Fifth, whole-brain values were calculated as the unweighted mean of regional segment values, based on the standard output of the analysis software. Although this approach may not fully reflect a voxelwise volume-weighted average, the impact is considered limited given the consistent findings across segments. Sixth, the supine and seated scans were acquired using two different CT systems. Furthermore, upright imaging is inherently more susceptible to motion artifacts compared to supine scanning, as maintaining absolute stillness against gravity is more challenging for participants. Although the upright system is based on the same detector and gantry configuration as the conventional 320-row CT system, subtle differences in system setup or processing, combined with the potential impact of minor involuntary movements in the seated position, may have influenced the measured perfusion values. Seventh, regional blood flow is strongly influenced by brain activity [37]. Humans are generally more alert in a seated than in a supine position, potentially leading to a functional increase in CBF while seated. Additionally, participants might have felt nervous or experienced intrusive thoughts during CT perfusion acquisition, possibly influencing regional brain perfusion. Lastly, more extreme upright postures, such as standing, may induce more pronounced gravitational effects than sitting [2, 9]. However, CT perfusion in the standing position was not feasible due to safety concerns including the risk of allergic reactions. Future studies may further explore these dynamic effects.

In conclusion, this study showed upright CTP detected posture-associated differences in perfusion metrics. Interindividual variability in perfusion responses to postural change was observed. These findings suggest that upright CT perfusion may serve as a valuable tool for investigating posture-related cerebral hemodynamics and interindividual variability under physiological gravitational conditions.

## Supplementary information

**Additional File 1: Fig. S1** Association between differences in diastolic and mean arterial pressure (seated minus supine) and the rate of change in perfusion parameters (seated *versus* supine position). **Fig. S2** Association between participant background parameters (age, height, and weight) and the rate of change in perfusion parameters (seated *versus* supine position). **Fig. S3** Individual changes in whole-brain cerebral perfusion parameters (cerebral blood flow (CBF), mean transit time (MTT), and cerebral blood volume (CBV)) during postural transition in Pattern I. Each line represents an individual participant, connecting perfusion values measured in the supine and seated positions. **Fig. S4** Individual changes in whole-brain cerebral perfusion parameters (cerebral blood flow (CBF), mean transit time (MTT), and cerebral blood volume (CBV)) during postural transition in Pattern II. Each line represents an individual participant, connecting perfusion values measured in the supine and seated positions.

## Data Availability

The datasets generated and/or analyzed during the current study are not publicly available due to institutional restrictions, as they can only be accessed within our laboratory. However, the data are available from the corresponding author on reasonable request, subject to on-site access at our institution.

## Abbreviations

CBF: Cerebral blood flow
CBV: Cerebral blood volume
CT: Computed tomography
CTP: Computed tomography perfusion
MAP: Mean arterial pressure
MTT: Mean transit time
PET: Positron emission tomography
