# Supplementary material for: Effect of body position on cerebral perfusion: a comparison of supine and seated positions assessed using conventional and upright CT perfusion
Source: Eur Radiol Exp. 2026 Jun 8;10:84. doi: 10.1186/s41747-026-00747-6 (PMC13246991; doi:10.1186/s41747-026-00747-6)
Supplement: Supplementary file 1 — Additional File 1: Fig. S1 Association between differences in diastolic and mean arterial pressure (seated minus supine) and the rate of change in perfusion parameters (seated versus supine position). Fig. S2 Association between participant background parameters (age, height, and weight) and the rate of change in perfusion parameters (seated versus supine position). Fig. S3 Individual changes in whole-brain cerebral perfusion parameters (cerebral blood flow (CBF), mean transit time (MTT), and cerebral blood volume (CBV)) during postural transition in Pattern I. Each line represents an individual participant, connecting perfusion values measured in the supine and seated positions. Fig. S4 Individual changes in whole-brain cerebral perfusion parameters (cerebral blood flow (CBF), mean transit time (MTT), and cerebral blood volume (CBV)) during postural transition in Pattern II. Each line represents an individual participant, connecting perfusion values measured in the supine and seated positions. [file 41747_2026_747_MOESM1_ESM.pdf]

Effect of body position on cerebral perfusion: a comparison of supine and seated positions assessed using conventional and upright CT perfusion

ELECTRONIC SUPPLEMENTARY MATERIAL

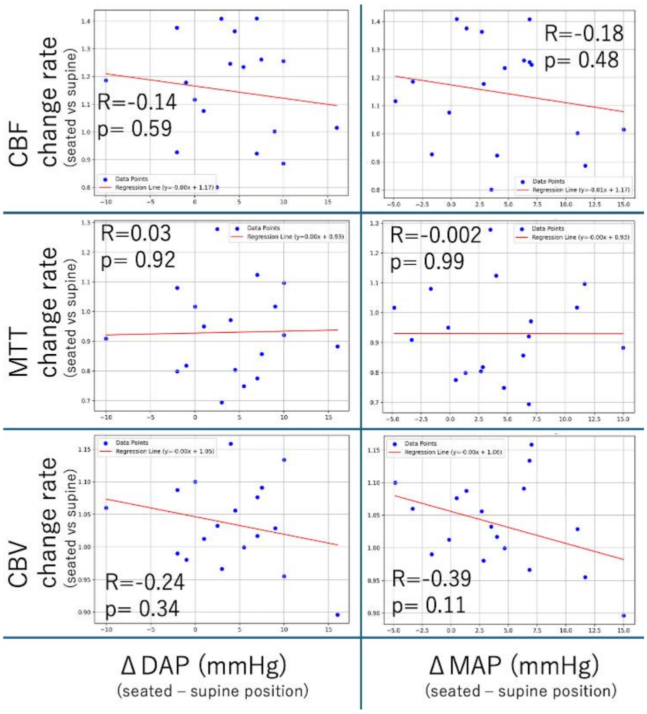

Fig. S1 Association between differences in diastolic and mean arterial pressure (seated minus supine) and the rate of change in perfusion parameters (seated *versus* supine position).

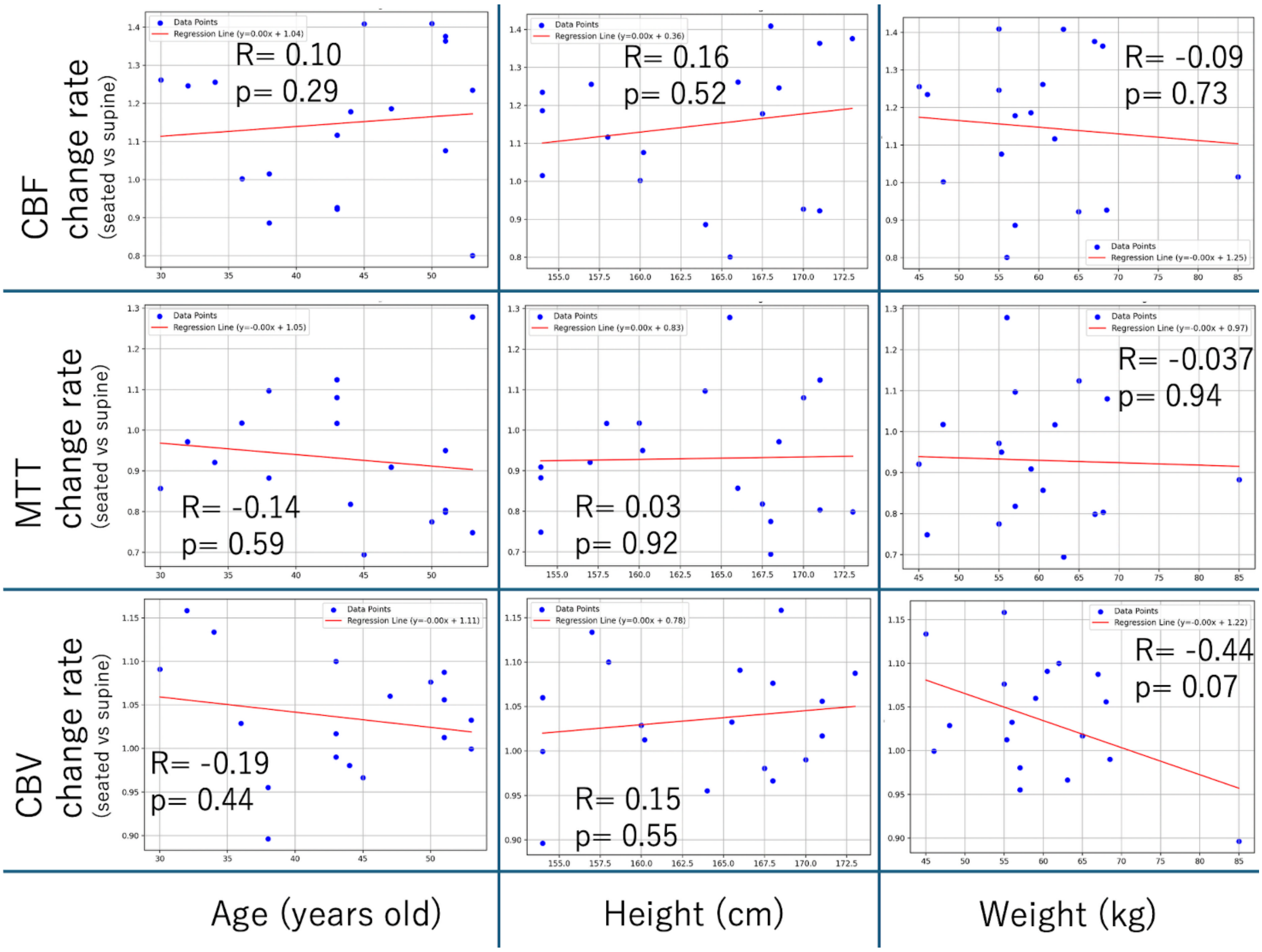

Fig. S2 Association between participant background parameters (age, height, and weight) and the rate of change in perfusion parameters (seated *versus* supine position).

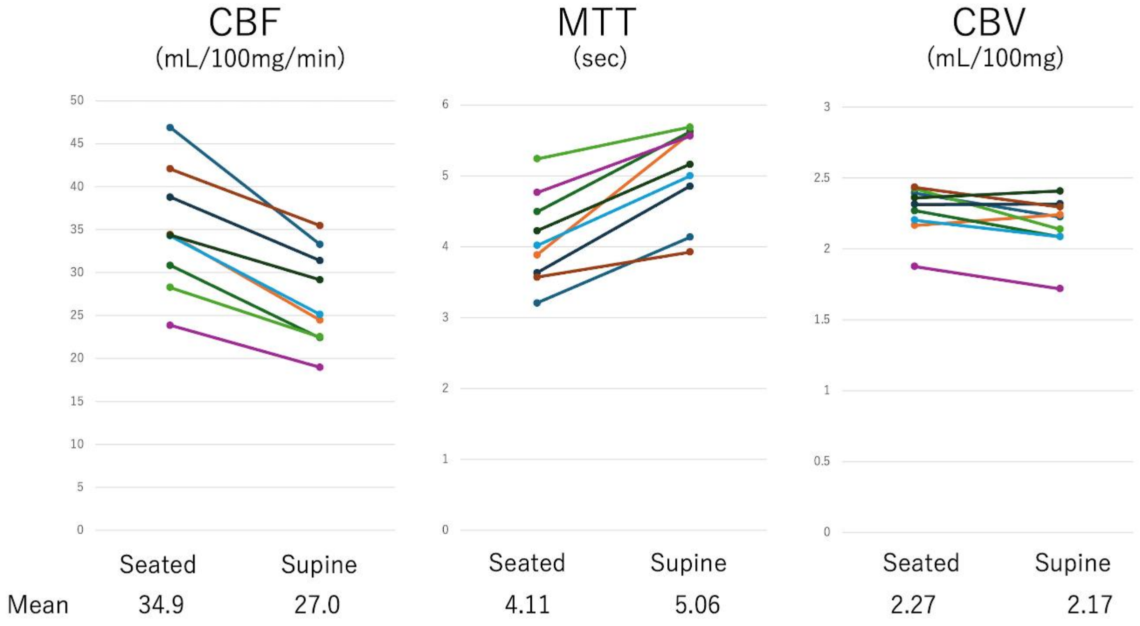

Fig. S3 Individual changes in whole-brain cerebral perfusion parameters (cerebral blood flow [CBF], mean transit time [MTT], and cerebral blood volume [CBV]) during postural transition in Pattern I. Each line represents an individual participant, connecting perfusion values measured in the supine and seated positions.

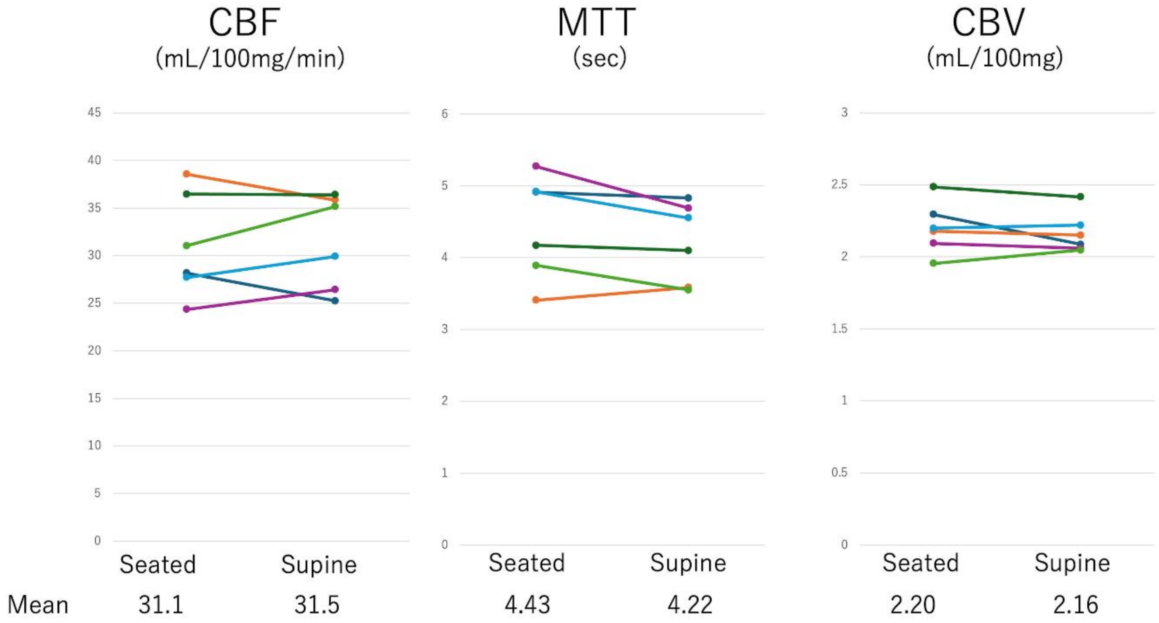

Fig. S4 Individual changes in whole-brain cerebral perfusion parameters (cerebral blood flow (CBF), mean transit time (MTT), and cerebral blood volume (CBV)) during postural transition in Pattern II. Each line represents an individual participant, connecting perfusion values measured in the supine and seated positions.
